# Antitumor effects of naturally occurring cardiac glycosides convallatoxin and peruvoside on human ER+ and triple-negative breast cancers

**DOI:** 10.1038/cddiscovery.2017.9

**Published:** 2017-02-27

**Authors:** Vivek Kaushik, Neelam Azad, Juan Sebastian Yakisich, Anand Krishnan V Iyer

**Affiliations:** 1 Department of Pharmaceutical Sciences, Hampton University School of Pharmacy, Kittrell Hall, Queen & Tyler Streets, Hampton, VA 23668, USA

## Abstract

Breast cancer is second most prevalent cancer in women, and the second only to lung cancer in cancer-related deaths. It is a heterogeneous disease and has several subtypes based on the presence or absence of hormone receptors and/or human epidermal growth factor receptor 2 (HER2). Hormone receptor-positive and HER2-enriched cancers can be targeted using hormone and HER2-targeting therapies such as trastuzumab or lapatinib. However, triple-negative breast cancers (TNBCs) do not express any of the receptors and therefore are resistant to most targeted therapies, and cytotoxic chemotherapies are the only viable option available for the treatment of TNBCs. Recently, cardiac glycosides (CGs) have emerged as potential anticancer agents that impart their antiproliferative effect by targeting multiple pathways. In this study our aim was to evaluate anticancer effects of two naturally occurring CGs, Convallatoxin (CT) and Peruvoside (PS), on ER+ and TNBCs cells. CT and PS demonstrated dose- and time-dependent cytotoxic effect on MCF-7 cells, which was further supported by loss of colony formation on drug treatment. CT and PS arrested MCF-7 cells in the G0/G1 phase and reduced the viability of MCF-7-derived mammospheres (MMs). Interestingly, while CT and PS imparted cell death in TNBCs cells from both Caucasians (MDA-MB-231 cells) and African Americans (MDA-MB-468 cells) in a dose- and time-dependent manner, the drugs were much more potent in MDA-MB-468 as compared with TNBC MDA-MB-231 cells. Both drugs significantly inhibited migration and invasion of both MCF-7 and MDA-MB-468 cells. An assessment of intracellular pathways indicated that both drugs were able to modulate several key cellular pathways such as EMT, cell cycle, proliferation and cell death in both cell types. Our data suggest a promising role for CGs in breast cancer treatment specifically in targeting TNBCs derived from African Americans, and provides impetus for further investigation of the anticancer potential of this class of drugs.

## Introduction

Breast cancer is the most common cancer in women, accounting for almost 29% of newly diagnosed cancer cases. According to http://seer.cancer.gov/ data, an estimated 246 660 new cases of breast cancer were reported in 2016, resulting in an estimated at 40 450 deaths, which account for 6.8% of cancer-related deaths in United States. Breast cancer is a heterogeneous disease and can be divided into five subtypes based on the expression of molecular markers such as the presence or absence of hormone (estrogen or progesterone) receptors (HR+/HR−) and excess levels of human epidermal growth factor receptor 2 (HER2+/HER2−) – Luminal A (HR+/HER2−), Luminal B (HR+/HER2+), HER2-enriched (HR−/HER2+), basal-like (almost 75% of this type of cancers belong to triple-negative (HR−/HER2−)) and normal breast-like tumors.^1–3^ Of all these subtypes, triple-negative breast cancer (TNBC) is the most aggressive cancer, and has much higher rates of relapse and shorter overall survival as compared with other subtypes. It is more common in premenopausal women and almost twice as common in African-American women as compared with Caucasian women in USA.^1,4,5^ Luminal A, Luminal B and HER2-enriched cancers can be targeted using hormone- and HER2-targeting therapies such as trastuzumab or lapatinib. However, there are no targeted therapies available for TNBCs due to lack of expression of molecular targets, and cytotoxic chemotherapy is the only treatment option available for TNBCs.^6^ For advanced disease TNBCs, several clinical trials are ongoing that use drugs that target angiogenesis, poly-ADP-ribose-polymerase (PARP), epidermal growth factor receptor (EGFR), phosoinositol-3 kinase, mitogen-activated protein kinase, checkpoint kinase and histone-deacetylase, but preliminary data suggest that the clinical benefit from such therapies was still limited.^6^ Therefore, identification, development and testing of new drugs that target breast cancers is of utmost importance for finding a permanent cure for this disease.

Cardiac glycosides (CGs) are a class of organic compounds consisting of a sugar (glycoside) and an aglycone (steroid) moiety. They are used for the treatment of heart ailments such as congestive heart failure, ischemia and cardiac arrhythmia. Interestingly, over the years, several reports have pointed towards potential anticancer activity of CGs. Digitoxin, digoxin, ouabain, oleandrin, bufalin etc. are some of the CGs that have been studied for their anticancer potential and have shown very potent anticancer effects in various types of cancers.^7–9^ However, concerns related to cardiotoxic side effects arising from their narrow therapeutic index rather prematurely dampened subsequent investigative efforts in delineating their cytotoxic potential against cancer. We recently designed a novel set of CG analogs that recapitulate the therapeutic benefits of Digitoxin signaling in cancer while overcoming Digitoxin-associated toxicity, and our preliminary study demonstrated potent antitumorigenic effects against several forms of cancer.^10^ This study showed that subtle changes in either sugar or steroid moiety can have telling effect on the cytotoxicity of the drug. For example, number of sugar units in the glycoside region, an oxygen vs nitrogen linkage between glycoside and aglycone and the number and position of different substituent such as hydrogen, hydroxyl or other groups, could significantly modify anticancer activity of the analogs. Following these key structural attributes of a potent CG, naturally occurring CGs Convallatoxin (CT), Peruvoside (PS) and Strophanthidin (ST) were selected for evaluation of their anticancer potential. Both CT and PS have a single sugar moiety but have differing substituents on glycoside as well as a aglycone unit, whereas ST has only aglycone unit (Supplementary Figure S1). We have already demonstrated their tumoristatic effects in multiple models of lung cancer, including routine culture and lung tumor spheres.^11^ Here in this study we have evaluated cytotoxic effects of CT and PS in breast cancer cells. To our knowledge, such a comparative analysis evaluating effects of CT and PS on breast cancer has not been performed before.

In this study, we first explored the anticancer role of CT, PS and ST in ER+ MCF-7 breast cancer cells. As seen in our previous study with lung cancer cells, both CT and PS inhibited MCF-7 cell viability in a dose- and time-dependent manner, whereas ST was largely inactive, probably due to the lack of sugar unit, which is one of the essential structural features for the anticancer efficacy of CGs. However, CT had much higher potency as compared with PS at both short- and long-term exposures. Furthermore, both CT and PS had a strong inhibitory effect on MCF-7 colony formation, and reduced the viability of MCF-7-derived mammospheres (MMs) in MCF-7 cells. This effect was cell-cycle-dependent and analysis of MCF-7 cells treated with CT and PS showed a G0/G1 phase cell cycle arrest. We assessed whether CT and PS exerted similar effects on TNBCs, and while both drugs inhibited cell viability in MDA-MB-231 and MDA-MB-468 cells, the potency of CT and PS was at least five times higher in MDA-MB-468 cells, and in a dose range equivalent to that observed in MCF-7 cells. We evaluated inhibition of cell migration and invasion of both MCF-7 and MDA-MB-468 cells, and CT and PS potently inhibited both effects. An evaluation of intracellular pathways in MCF-7 and MDA-MB-468 cells in response to both CT and PS demonstrated that both drugs were capable of impacting multiple pathways related to EMT, proliferation and cell death in both cell lines. Our data suggest an important role for CT and PS as potential therapeutic strategies for the treatment of breast cancer; more importantly towards TNBCs derived from African-American patients due to the increased susceptibility of MDA-MB-468 cells.

## Results

### CT and PS exert cytotoxic effects in ER+ breast cancer MCF-7 cells

**Figure 1:**
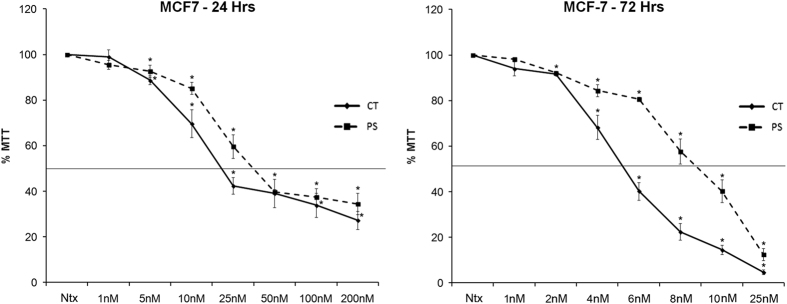
CT and PS inhibit the viability of ER+ MCF-7 breast cancer cells in a dose- and time-dependent manner. MCF-7 cells were incubated in serum-free media with indicated doses of CT and PS for 24 (left panel) or 72 h (right panel). Cell viability was assessed by the MTT assay. Plots show mean values±S.E. of quadruplicates determinations of three or more experiments at *P*<0.05.

In order to assess cytotoxicity of drugs, MCF-7 cells were treated with various doses of CT, PS or ST for 24 h, and assessed for cytotoxic effects using the MTT assay. Both CT and PS reduced cell viability in a dose-dependent manner, while ST showed little effect. In order to determine IC_50_ values at longer exposure times, MCF-7 cells were treated with increasing doses of CT or PS for 72 h. The IC_50_ values for CT and PS in MCF-7 cells were 27.65±8.5 and 47.5±13.1 nM, respectively, at 24 h and 5.32±0.15  and 9.4±0.57 nM, respectively, at 72 h (Figure 1).

### CGs inhibit colony formation in MCF-7 cells

**Figure 2:**
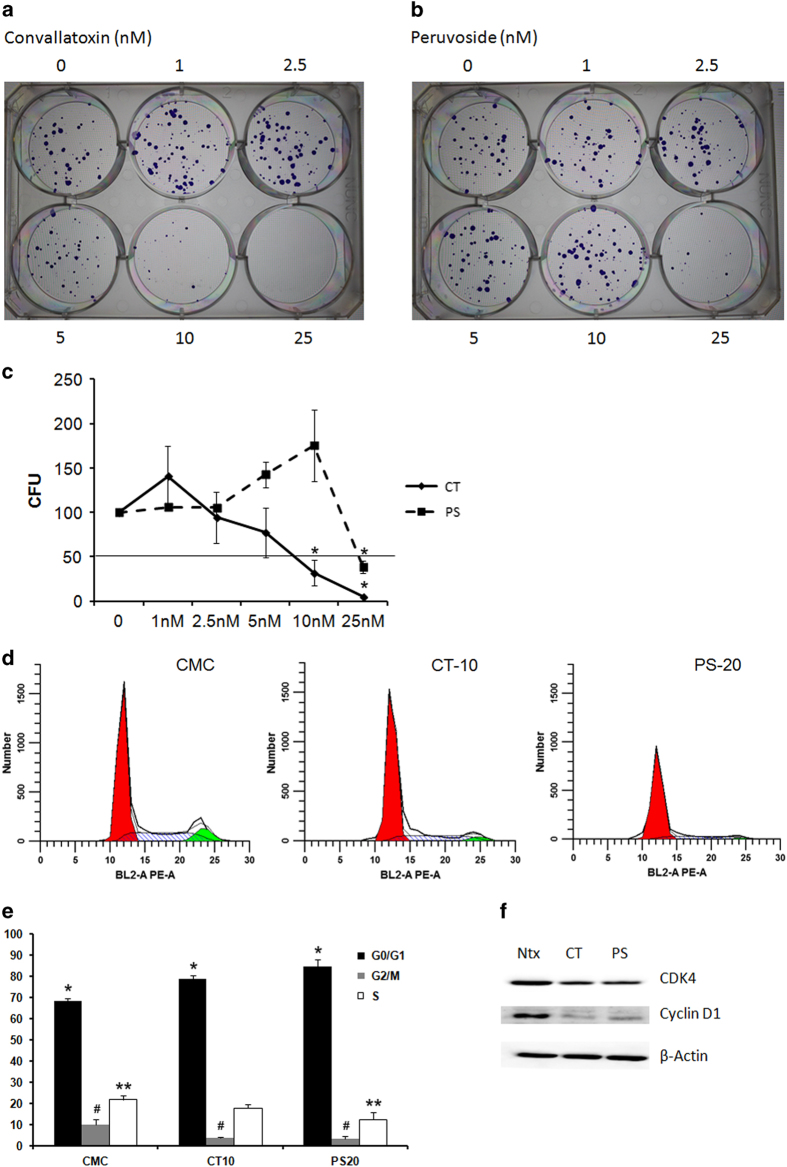
CT and PS prevent MCF-7 colony formation and arrest cell cycle in the G0/G1 stage. MCF-7 cells were plated (200 cells/well), allowed to adhere overnight, treated with the indicated concentrations of drugs for 72 h in serum-free media. After treatment, cells were allowed to form colony in media containing serum (10%) for 7–10 days. Representative images of colonies (**a**) CT treatment, (**b**) PS treatment. (**c**) Dose–response curves for CT and PS. (**d**) Cells were synchronized in the G0/G1 phase by incubating in serum-free media for 72 h followed by drug treatments in complete media for 24 h. Cells were fixed with 70% ethanol for at least 24 h at −20 °C, stained with propidium iodide and analyzed by flow cytometry. (**e**) Quantitative representation of cell cycle data. (**f**) Western blot analysis of cell cycle regulating proteins CDK4 and Cyclin D1. Data are the mean values ±S.E. of at least three independent experiment performed in triplicates at *P*<0.05.

We evaluated effect of CT and PS on the ability of MCF-7 cells to form colonies. MCF-7 cells were exposed to increasing doses of CT (0–25 nM) and PS (0–25 nM) and assessed for formation of colonies. While both the drugs inhibited colony formation in a dose-dependent manner, CT was much more potent as compared with PS (Figures 2a and b), which recapitulated the results obtained using the MTT assay. From colony-forming assay results, we estimated that CT had an IC_50_ of 6.43±1.72 nM while PS was less effective, with an IC_50_ of roughly 25 nM (Figure 2c).

### CT and PS arrest MCF-7 cells in the G0/G1 phase

Since both CT and PS inhibited cell viability and colony formation in MCF-7 cells, we assessed whether this effect was mediated by effects on cell cycle. Breast cancer cells were synchronized in the G0/G1 phase by serum starvation for 72 h, and then treated with CT 10 nM and PS 20 nM for 24 h in complete media. While cells released into the S-phase from G0/G1 in control treatments, both drugs induced arrest of more than 80% of cells in the G0/G1 phase. The arrest in the G0/G1 phase was at the expense of decrease in cells in the S and G2/M phases (Figures 2d and e). In order to validate and assess this effect, we probed for important proteins that mediate cell cycle progression including CDK4 and cyclin D1. Both CT and PS significantly inhibit the expression of cell cycle proteins (Figure 2f).

### CT and PS decrease cell viability of MCF-7 cells growing as floating MSs

**Figure 3:**
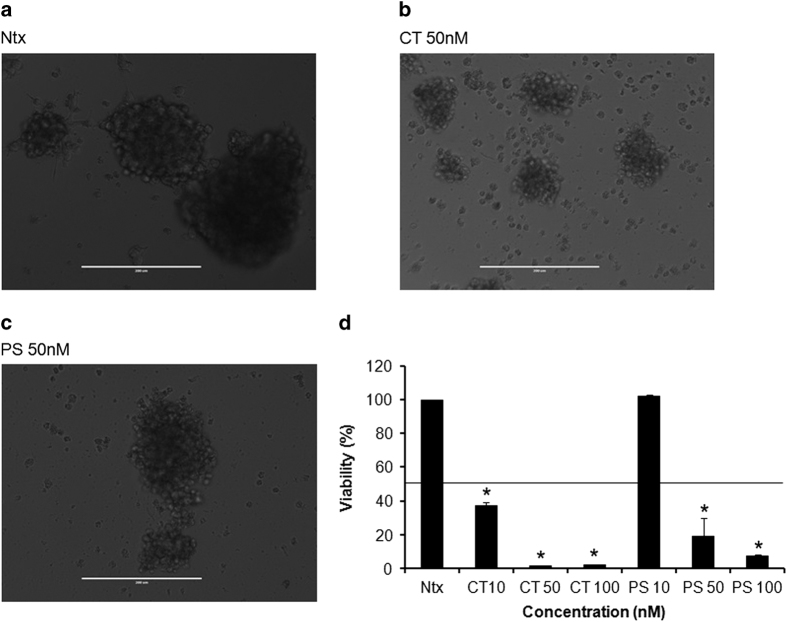
CT and PS decrease viability of breast cancer mammosphers (MSs). MSs were generated as described in the Material and Methods section. Representative images of treated MSs clearly demonstrates reduction of viability of MSs on drug treatment: (**a**) Untreated (Ntx), (**b**) CT 50 nM, (**c**) PS 50 nM. (**d**) Dose–response of CT and PS on MSs was measured by CCK-8 essay. Data are the mean values±S.E. of at least three independent experiment performed in triplicates at *P*<0.05.

The ability of CT and PS to inhibit the viability of cancer cells with increased stemness was evaluated in MSs. CT at concentrations lower than 10 nM could significantly decrease the viability of MSs, consistent with our previous experiments; PS was much less effective, showing inhibitory effect at concentrations much higher than 10 nM (Figures 3a–d).

### CT and PS show selective inhibition of MDA-MB-468 over MDA-MB-231 TNBC cells

**Figure 4:**
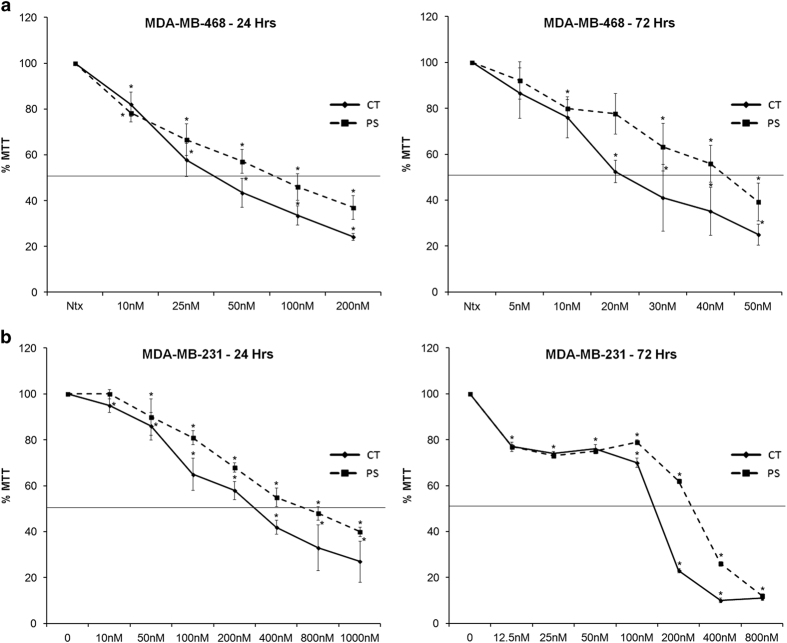
CT and PS inhibit the viability of triple-negative breast cancer cells (TNBCs) MDA-MB-231 and MDA-MB-468 cells in a dose and time-dependent manner. TNBCs cells were incubated in serum-free media with indicated doses of CT and PS for 24 or 72 h, respectively, and cell viability was assessed by the MTT assay. (**a**) MDA-MB-468 cells and (**b**) MDA-MB-231 cells. Plots show mean values±S.E. of quadruplicates determinations of three or more experiments at *P*<0.05.

Since CT and PS demonstrated increased inhibition of MSs, we evaluated cytotoxic effect of these drugs in TNBCs. Both MDA-MB-231 (Caucasian) and MDA-MB-468 (African-American) TNBC cells were treated with various doses of CT, PS or ST for 24 h, and assessed for cytotoxic effects using the MTT assay (Figures 4a and b; Supplementary Figure S2). Both CT and PS reduced cell viability in a dose-dependent manner, while ST showed little effect. In order to determine IC_50_ values at longer exposure times, TNBC cells were treated with increasing doses of CT or PS for 72 h (Figures 4a and b). Interestingly, both CT and PS showed much higher selectivity towards MDA-MB-468 cells rather than MDA-MB-231 cells, as reflected by a seven–eight-fold lower IC_50_ dose for MDA-MB-468 cells at 24 and 72 h. The IC_50_ values for CT and PS in MDA-MB-468 cells were 41.30±6.33  and 79.87±2.19 nM, respectively, at 24 h and 22.90±1.10 and 44.66±5.14 nM, respectively, at 72 h. The IC_50_ values for CT and PS in MDA-MB-231 cells were 281.03±36.01  and 565.08±56.47 nM at 24 h and 155.55±28.49  and 308.05±56.39 nM at 72 h.

### CT and PS inhibit cell migration and invasion of breast cancer cells

**Figure 5:**
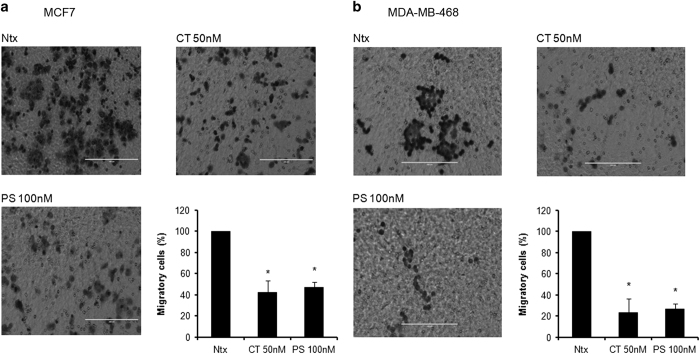
CT and PS inhibit migration of MCF-7 and MDA-MB-468 breast cancer cells. Representative images (×20) of migratory cells in (**a**) MCF-7 and (**b**) MDA-MB-468 cells after treatment with the indicated concentration of Convallatoxin (CT) or Peruvoside (PS). The plot shows the mean values±S.E. of migratory cells from five pictures each taken under a light microscope for two independent experiments at *P*<0.05.

**Figure 6:**
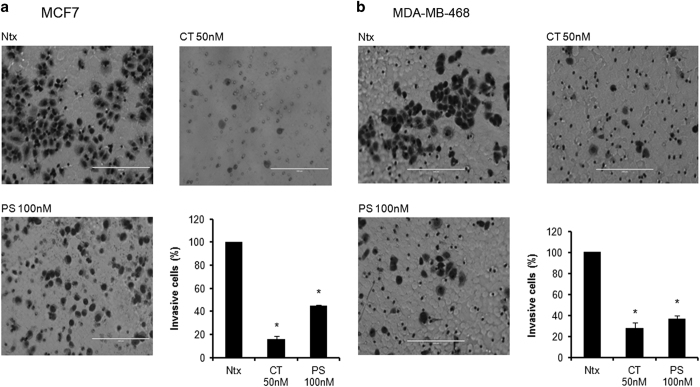
CT and PS inhibit invasion of MCF-7 and MDA-MB-468 breast cancer cells. Representative images (×20) of invasive cells in (**a**) MCF-7 and (**b**) MDA-MB-468 cells after treatment with the indicated concentration of Convallatoxin (CT) or Peruvoside (PS). The plot shows the mean values ±S.E. of invasive cells from five pictures each taken under a light microscope for two independent experiments at *P*<0.05.

We also assessed for inhibitory effects of CT and PS on phenotypic behavior of both types of breast cancer cells. Trans-cell migration and invasion assays were performed using breast cancer cells in the presence of CT (50 nM) and PS (100 nM). While both CT and PS significantly inhibited migration (Figures 5a and b) and invasion (Figures 6a and b) in both MCF-7 and MDA-MB-468 cells, CT was much more potent as compared with PS.

### CT and PS exert changes on key biochemical pathways in MCF-7 and MDA-MB-468 cells

Since both CT and PS clearly exert antitumor effects in breast cancer cells, we delineated some of the important pathways that contribute to such effects. Cells were treated with CT and PS for 24 h, and assayed for proteins related to various biochemical pathways that can impact tumorigenesis and cancer progression, including EMT, proliferation and cell death in both MCF-7 and MDA-MB-468 cells. Our data demonstrated that while both CT and PS exerted similar effects on a wide range of proteins across pathways in both cell lines, there were few proteins which were differentially regulated by CT and PS in a cell line specific manner.

**Figure 7:**
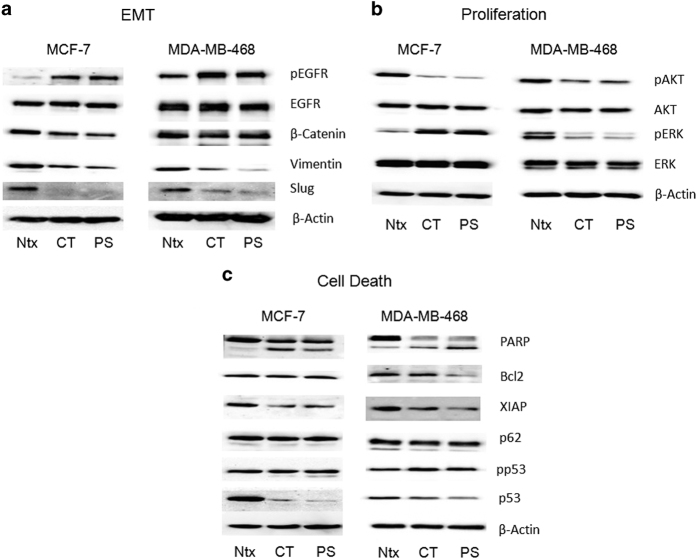
CT and PS modulate the expression of key signaling pathways. Cell lysates (30 *μ*g) collected after 24 h of drug treatment were analyzed for the indicated proteins within the (**a**) EMT, (**b**) proliferation and (**c**) cell death pathways by western blotting. Blots were re-probed for *β*-actin to confirm equal loading of samples. Representative blots from three independent experiments are shown.

We assayed for effect of CT and PS on EMT markers such as EGFR, pEGFR, *β*-catenin, Vimentin and Slug. After 24-h treatment, both CT and PS significantly inhibited all the proteins (Figure 7a). Assessment of canonical cell proliferation pathways including Akt and ERK demonstrated that while p-Akt was downregulated in both the cell lines, p-ERK was differentially regulated, being activated in MCF-7 cells and inhibited in MDA-MB-468 cells (Figure 7b). Expression of pro-apoptotic proteins such as cleaved PARP and phospho-p53 increased upon treatment with drugs, while levels of the cell-survival proteins Bcl2 and XIAP decreased upon treatment with CT and PS. Expression of p62, an important autophagy marker, did not change upon drug treatment (Figure 7c).

## Discussion

CGs are an emerging class of potential anticancer drugs that exert antiproliferative activity in a variety of cancers at very low concentrations.^12,13^ Although CGs exert their antitumor effects at very low concentrations, they have lethal cardiotoxic side effects which limits their therapeutic use to a range of roughly 40–50 nM.^14–16^ Digitoxin, ouabain, oleandrin and proscillaridin are some of CGs which have shown anticancer properties in breast cancer.^17^ In a recent study, we have demonstrated the anticancer potential of two naturally occurring CG CT and PS in human lung cancer H460 cells growing in routine culture conditions as well serum-starved lung tumor spheres.^11^ These drugs demonstrated potent anticancer efficacy within therapeutic range of ~50 nM. These compounds have never been explored in the treatment of breast cancer.

First, we evaluated the effect CT, PS and ST (Figure 1 and Supplementary Figure S2) on cell viability using MTT assay for ER+ MCF-7 cells. While ST was largely inactive, CT and PS potently inhibited (CT was more active than PS) the viability of MCF-7 cells in a dose and time-dependent manner. ST is similar to Digitoxigenin, a prototypical CG that lacks the sugar moiety.^18,19^ ST was a weak inhibitor of proliferation when compared with CT or PS.^18^ On the other hand, CT and PS that contain one sugar residue were much stronger inhibitors, suggesting that the sugar moiety is important for conferring anticancer activity. Interestingly, any further increase in the number of sugars decreased activity.^10^ The results obtained using the MTT assay were recapitulated by the colony-forming assay that showed a similar trend in the ability of these drugs to inhibit the clonogenicity of MCF-7 cells (inhibitory effect: PS<CT; Figures 2a–c).

Cytotoxic effects of several anticancer drugs are associated with cell cycle arrest. Cell cycle analysis by flow cytometry showed that both CT and PS (10 and 20 nM) arrested cell cycle progression by increasing the percentage of cells in the G0/G1 phase in MCF-7 cells (Figures 2d and e). This result was confirmed by western blot analysis that showed reduced levels of CDK4 and Cyclin D1 in CT and PS treated cells for 24 h (Figure 2f). Similar effects have been observed with other plant-based drugs. For example, Salidroside found in the plant *Rhodiola rosea* arrested MCF-7 cells in the G0/G1 phase.^20^ Root extract of *Glehnia littoralis*, which is an oriental herbal cough medicine has been shown to arrest cell cycle in the G0/G1 phase in MCF-7 cells.^21^ Additionally, anticancer potential of both the drugs was also evaluated in more resistant floating MSs of MCF-7 cells using CCK-8 assay (Figure 3). As expected, CT was much more active than PS, as a 10-nM dose of CT significantly inhibited MSs viability while similar dose of PS had no effect.

TNBCs are the most aggressive and resistant type of breast cancers with a very high relapse rate. Currently, there are no specific anticancer therapeutic agents available for the treatment of TNBCs, and current treatments rely on nonspecific cytotoxic chemotherapies.^6^ Therefore, identification, evaluation and development of new drugs which can target TNBCs is extremely important for effective and permanent cure of TNBCs. As CT and PS demonstrated very potent cytotoxicity in both MCF-7 cells and the more resistant MCF-7-derived MSs, we assessed their anticancer efficacy in more aggressive and resistant TNBCs. Anticancer potential of these drugs was evaluated in MDA-MB-231 (Caucasian) and MDA-MB-468 (Africa-American) TNBC cells by MTT assay (Figure 4). Both CT and PS reduced cell viability in both types of TNBCs and as expected CT was more potent than PS. However, both the drugs exhibited much more selectivity towards MDA-MB-468 TNBC cells (Figure 4a) then MDA-MB-231 TNBC cells (Figure 4b), with at least a seven-fold lower IC_50_ values. Since, IC_50_ values for both the drugs far exceeded than the tolerable levels in MDA-MB-231 cells, only MDA-MB-468 cells were pursued for further evaluation.

Cancer metastasis is responsible for spread of this disease to different parts of the body. An effective anticancer drug must stop spread of cancer by curbing migration and invasion of cancer cells. Both CT and PS inhibited the migratory (Figures 5a and b) and invasive (Figures 6a and b) properties of MCF-7 and MDA-MB-468 cells. At a molecular level, both CT and PS downregulated the expression of proteins associated with the EMT phenotype (Figure 7a) which are associated with cancer stem cell phenotypes that promotes tumorigenesis, metastasis and chemoresistance.^22^ Therefore, reduced migratory and invasive ability of breast cancer cells on drug treatment may be attributed to downregulation of EMT proteins.

Both CT and PS exerted a profound inhibitory effect on viability, colonogenity, migration and invasion of breast cancer cells. Drug treatments in MDA-MB-468 cells however failed to arrest TNBCs in any specific cell cycle phase (data not shown). A detailed western blot analysis of key pathways was performed to gain insight in the mechanistic aspect of CT and PS treatment in breast cancer cells. Effect of CGs treatment was assessed on several cell proliferation markers. CT and PS significantly reduced phospho-Akt expression in both cell types. However, phospho-ERK expression was differentially regulated depending on cell type, while phospho-ERK expression was upregulated in MCF-7 cells, it was downregulated in MDA-MB-468 cells. Similar differential expression has been observed for doxorubicin treatments in MCF-7 and MDA-MB-231 cells.^23^ Cucurbitacin E, which is a biochemical compound from the family of Cucurbitacins, downregulates phospho-ERK expression in MDA-MB-468 cells.^24^

Reduction in XIAP expression and up regulation of cleaved PARP on drug treatment indicates an apoptotic cell death. Both CT and PS inhibited XIAP level and increase PARP cleavage in both cell lines. Bcl2 is a procell-survival protein which prevents cell apoptosis by inhibiting release of cytochrome *c* from the mitochondria.^25^ Drug treatment resulted in downregulation of Bcl2 in TNBCs; however, it was not as evident in MCF-7 cells.

p53 is a tumor suppressor protein which gets activated in response to DNA damage and gene mutations and results in cell cycle arrest, DNA repair or apoptosis.^26^ The active form of p53 is phosphorylated p53 (phospho-p53). In cells, p53 levels are regulated by a suppressor protein MDM2, which targets p53 for ubiquitination and proteasomal degradation.^27^ MDM2 has a reduced interaction with phospho-p53, and its accumulation promotes apoptosis.^28^ There was a significant increase in phospho-p53 expression in MDA-MB-468 cells; however, phospho-p53 expression remained unchanged in MCF-7 cells. Basal p53 expression showed a consistent and significant decrease in both the cell lines upon drug treatment. On the other hand, there was no change in the expression of the autophagy marker p62 upon drug treatment, indicating autophagy independent cell death.

Overall, CT and PS exhibited a very potent cytotoxic potential towards breast cancer cells by inhibiting cell viability, colonogenicity, cell invasion and migration at nanomolar concentrations. They imparted these effects by modulating several key cellular pathways. Interestingly, both CT and PS showed a very high selectivity towards TNBC cell lines derived from African-American patients. This is of importance in general, as TNBCs are very aggressive and most resistant breast cancers which lack specific targeted therapies; more so in African-American women who are inherently more susceptible to have TNBCs.

## Materials and Methods

### Chemicals and reagents

Convallatoxin was obtained from Pfaltz & bauer (Waterbury, CT, USA). Peruvoside was purchased from Research Plus, Inc. (Barnegat, NJ, USA). Strophanthidin, Thiazolyl blue tetrazolium bromide (MTT), Cisplatin, Sodium dichromate (Na_2_Cr_2_O_7_.2H_2_O) [Cr(VI)], Triton X-100, DNAse free RNAse and Propidium Iodide were obtained from Sigma Aldrich (St Louis, MO, USA). Antibodies for p53, phospho-p53, CDK4, Cyclin D1, PARP, XIAP, p-62, phospho-Akt (Ser 473), total-Akt, phospho-ERK, total-ERK, pEGFR, EGFR, *β*-Catenin, Vimentin, Slug and peroxidase-labeled secondary rabbit and mouse antibodies were obtained from Cell Signaling Technology (Denvers, MA, USA). Antibody for Bcl2 was procured from Santa Cruz Biotechnology (Dallas, TX, USA). Bicinchoninic acid and Supersignal West Pico chemiluminescent substrate was purchased from Thermo Fisher Scientific (Waltham, MA, USA).

### Preparation of anticancer drugs

CT, PS and ST were stored as stock solution (10 mM) in DMSO in glass containers at −20 °C. Final dilutions were prepared freshly in culture media before use. The control experiment contained the highest concentration (0.001%) of DMSO only.

### Cell culture

The human breast cancer cell lines MCF-7, MDA-MB-231 and MDA-MB-468 were obtained from American Type Culture Collection (Manassas, VA, USA). Cells were cultured in DMEM/high glucose supplemented with 10% FBS, 2 mM l-glutamine, 100 U/ml penicillin, and 100 mg/ml streptomycin. All cells were cultured in a 5% CO_2_ environment at 37 °C.

### MTT assay

Antiproliferative effect of CT, PS and ST on breast cancer cells was determined by performing MTT assay. Roughly, 10 000 cells per well were plated in 96-cell culture microplate (Corning Inc., Corning, NY, USA) and incubated overnight in full media (DMEM containing 10% FBS) for cells to adhere to plate. Cells were then treated with various concentrations of drugs and vehicle for 24 and 72 h in serum-free media. Cell viability was evaluated by the MTT (Sigma Aldrich) assay. The absorbance of solubilized formazan was read at 570 nm using ELISA reader (Bio-TEK, Synergy-1, Winooski, VT, USA).

### Colony-forming assay

Colony-forming assay was performed as previously described.^29^ Briefly, 200 cells/well were plated in six-well plates and allowed to adhere overnight in complete media (DMEM containing 10% FBS). Cells were then treated with drugs at the indicated concentration or with vehicle alone for 72 h in serum-free media. After treatment cells were incubated with complete media for 7–10 days (media was changed every 72 h). Then cells were fixed with 3.7% formaldehyde for 60 min, stained with 0.01% crystal violet and photographed. Colonies were counted using ImageJ software (ImageJ Ver. 1.48, http://imagej.nih.gov/ij/).

### Cell cycle analysis

Cell cycle analysis based on DNA content was performed as follows. Approximately, 500 000 cells were plated in a six-well plate in 0.5% FBS DMEM media overnight (1% for MDA-MB-468). This media was removed next day and cells were synchronized in the G0/G1 phase by serum starvation for next 72 h, followed by 24 h treatment with different concentrations of drugs in complete media (DMEM 10% FBS). Cells were then trypsinized and centrifuged at 14 000 g for 8 min to make a cell pellet. Supernatant was removed and cells were resuspended and centrifuged twice in PBS. This cell pellet was then fixed in 1 ml of 70% ethanol for at least 24 h at −20 °C. Cells were centrifuged and ethanol was removed. These cells were then washed and centrifuged twice in PBS followed by resuspension of cells in 0.4 ml propidium iodide staining solution. The cells were then analyzed with FACScan (Becton Dickinson Immunocytometry, Mountain View, CA, USA) and ModFit LT cell cycle analysis software (Verity Software, Topsham, ME, USA).

### Mammospheres

MSs were generated as previously described.^30^ Briefly, cells grown in CM (70-80% confluency) were cultured overnight in serum-free media (SFM, same as CM but without FBS). Then cells were trypsinized and incubate in SFM for>14 days in poly-HEMA coated plated to prevent attachment. MSs grown in SFM for >14 days were used for subsequent experiments. For viability assay, MSs were collected in 15-ml Falcon tubes, centrifuged at 700 r.p.m.×3 min and resuspended in fresh SFM. In order to plate the same number of cells, this cells suspension was split in 1 ml aliquots. Vehicle (DMSO) or drugs were added to each aliquot and then 150 *μ*l cell suspension was loaded into each microwell (in a 96-well plate) and incubated for 72 h. Viability was measured using the CCK-8 assay.

### CCK-8 assay

Cell viability was evaluated by the CCK-8 assay (Dojingo Laboratories, Rockville, MD, USA) that uses the water-soluble tetrazolium salt, WST-8 as follows: 15 *μ*l of WST-8 solution was added to each microwell, incubated for 60–120 min and the absorbance was read at 450 nm using Gen 5 2.0 All-In-One microplate reader (Bio-TEK, Instruments Inc.). In parallel, microwells incubated with media alone followed by incubation with WST-8 solutions for equivalent time points were used to subtract background levels for the accurate analysis of cells viability.

### Migration and invasion assays

Migration and invasion assays were performed using overnight serum-starved cells. For migration assays breast cancer cells (1.05×10^5^ cells) were seeded in 8-*μ*m pore size Transwell Inserts (Corning Inc.) in DMEM (without FBS) without drugs (DMSO as control) or with CT (50 nM) or PS (100 nM). Inserts were placed in 24-well plates with DMEM containing 10% FBS in the presence of DMSO (Control) or CT (50 nM) or PS (100 nM). After 24 h, cell migration was quantified. Non-migratory cells were removed by gently removing the media and washing the cells with PBS. The membranes containing migratory cells were stained with Hema 3 Stat Pack (Fisher, Kalamazoo, MI, USA) following the manufacturer’s instructions. Migratory cells were photographed (five pictures for each treatment) under a light microscope at ×20 objective and counted. Invasion assays were performed in the similar way except that cells were seeded in Matrigel coated 8-*μ*m pore size Transwell Inserts (Corning Inc.) and invasion was quantified after 48 h. All experiments were independently repeated three times.

### Western blot analysis

After specific treatments, cells were harvested and lysed on ice for 30 min in lysis buffer containing 150 mM NaCl, 100 mM Tris (pH 8.0), 1% Triton X-100, 1% deoxycholic acid, 0.1% SDS, 5 mM EDTA, 10 mM sodium formate, 1 mM sodium orthovanadate, 2 mM leupeptin, 2 mM aprotinin, 1 mM phenylmethylsulfonyl fluoride, 1 mM dithiothreitol and 2 mM pepstatin A. After centrifugation at 14 000* g* for 15 min at 4 °C, the supernatant was collected as the total cellular protein extract. The protein concentrations were determined using a bicinchoninic acid protein assay kit (Pierce Biotechnology, Rockford, IL, USA). An equal amount of proteins per sample (30 *μ*g) was resolved on a 10% sodium dodecyl sulfate-polyacrylamide gel by electrophoresis (SDS-PAGE) and transferred onto a nitrocellulose membrane. The membrane was blocked with T-PBS (0.3% Tween-20 in PBS) containing 5% dry milk and incubated with primary antibody overnight at 4 °C. After three washes with T-PBS, the membrane was incubated with HRP-conjugated secondary antibody for 2 h at room temperature and then washed with 0.05% Tween-20 in PBS. Immunoreactive proteins were detected by chemiluminescence (Supersignal West Femto; Pierce) and quantified by imaging densitometry using UN-SCAN-IT digitizing software (Silk Scientific, Orem, UT, USA). Mean densitometry data from independent experiments were normalized to results in cells from control experiments.

## References

[bib1] Carey LA, Perou CM, Livasy CA, Dressler LG, Cowan D, Conway K et al. Race, breast cancer subtypes, and survival in the Carolina Breast Cancer Study. JAMA 2006; 295: 2492–2502. 16757721 10.1001/jama.295.21.2492

[bib2] Perou CM, Sorlie T, Eisen MB, van de Rijn M, Jeffrey SS, Rees CA et al. Molecular portraits of human breast tumours. Nature 2000; 406: 747–752. 10963602 10.1038/35021093

[bib3] Sørlie T, Perou CM, Tibshirani R, Aas T, Geisler S, Johnsen H et al. Gene expression patterns of breast carcinomas distinguish tumor subclasses with clinical implications. Proc Natl Acad Sci USA 2001; 98: 10869–10874. 11553815 10.1073/pnas.191367098 PMC58566

[bib4] Bauer KR, Brown M, Cress RD, Parise CA, Caggiano V. Descriptive analysis of estrogen receptor (ER)-negative, progesterone receptor (PR)-negative, and HER2-negative invasive breast cancer, the so-called triple-negative phenotype: a population-based study from the California Cancer Registry. Cancer 2007; 109: 1721–1728. 17387718 10.1002/cncr.22618

[bib5] Stead LA, Lash TL, Sobieraj JE, Chi DD, Westrup JL, Charlot M et al. Triple-negative breast cancers are increased in black women regardless of age or body mass index. Breast Cancer Res 2009; 11: R18. 19320967 10.1186/bcr2242 PMC2688946

[bib6] Palma G, Frasci G, Chirico A, Esposito E, Siani C, Saturnino C et al. Triple negative breast cancer: looking for the missing link between biology and treatments. Oncotarget 2015; 6: 26560–26574. 26387133 10.18632/oncotarget.5306 PMC4694936

[bib7] Bielawski K, Winnicka K, Bielawska A. Inhibition of DNA topoisomerases I and II, and growth inhibition of breast cancer MCF-7 cells by ouabain, digoxin and proscillaridin A. Biol Pharm Bull 2006; 29: 1493–1497. 16819197 10.1248/bpb.29.1493

[bib8] Kumar A, De T, Mishra A, Mishra AK. Oleandrin: a cardiac glycosides with potent cytotoxicity. Pharmacogn Rev 2013; 7: 131–139. 24347921 10.4103/0973-7847.120512 PMC3841991

[bib9] Yin PH, Liu X, Qiu YY, Cai JF, Qin JM, Zhu HR et al. Anti-tumor activity and apoptosis-regulation mechanisms of bufalin in various cancers: new hope for cancer patients. Asian Pac J Cancer Prev 2012; 13: 5339–5343. 23317181 10.7314/apjcp.2012.13.11.5339

[bib10] Iyer AK, Zhou M, Azad N, Elbaz H, Wang L, Rogalsky DK et al. A direct comparison of the anticancer activities of digitoxin MeON-neoglycosides and O-glycosides: oligosaccharide chain length-dependent induction of caspase-9-mediated apoptosis. ACS Med Chem Lett 2010; 1: 326–330. 21103068 10.1021/ml1000933 PMC2916185

[bib11] Kaushik V, Yakisich JS, Azad N, Kulkarni Y, Venkatadri R, Wright C et al. Anti-tumor effects of cardiac glycosides on human lung cancer cells and lung tumorspheres. J Cell Physiol 2016; e-pub ahead of print 23 September 201610.1002/jcp.25611. 10.1002/jcp.25611 27662422

[bib12] Calderón-Montaño JM, Burgos-Morón E, Orta ML, Maldonado-Navas D, García-Domínguez I, López-Lázaro M. Evaluating the cancer therapeutic potential of cardiac glycosides. Biomed Res Int 2014; 2014: 794930. 24895612 10.1155/2014/794930 PMC4033509

[bib13] Slingerland M, Cerella C, Guchelaar HJ, Diederich M, Gelderblom H. Cardiac glycosides in cancer therapy: from preclinical investigations towards clinical trials. Invest New Drugs 2013; 31: 1087–1094. 23748872 10.1007/s10637-013-9984-1

[bib14] Einbond LS, Wu HA, Su T, Chang T, Panjikaran M, Wang X et al. Digitoxin activates EGR1 and synergizes with paclitaxel on human breast cancer cells. J Carcinog 2010; 9: 10. 21139994 10.4103/1477-3163.72578 PMC2997271

[bib15] Kometiani P, Liu L, Askari A. Digitalis-induced signaling by Na+/K+-ATPase in human breast cancer cells. Mol Pharmacol 2005; 67: 929–936. 15602003 10.1124/mol.104.007302

[bib16] López-Lázaro M, Pastor N, Azrak SS, Ayuso MJ, Austin CA, Cortés F. Digitoxin inhibits the growth of cancer cell lines at concentrations commonly found in cardiac patients. J Nat Prod 2005; 68: 1642–1645. 16309315 10.1021/np050226l

[bib17] Elbaz HA, Stueckle TA, Tse W, Rojanasakul Y, Dinu CZ. Digitoxin and its analogs as novel cancer therapeutics. Exp Hematol Oncol 2012; 1: 4. 23210930 10.1186/2162-3619-1-4 PMC3506989

[bib18] Schneider NF, Geller FC, Persich L, Marostica LL, Pádua RM, Kreis W et al. Inhibition of cell proliferation, invasion and migration by the cardenolides digitoxigenin monodigitoxoside and convallatoxin in human lung cancer cell line. Nat Prod Res 2015; 7: 1–5. 10.1080/14786419.2015.1055265 26252521

[bib19] Zhang J, Ponomareva LV, Nandurkar NS, Yuan Y, Fang L, Zhan CG et al. Influence of sugar amine regiochemistry on digitoxigenin neoglycoside anticancer activity. ACS Med Chem Lett 2015; 6: 1053–1058. 26487911 10.1021/acsmedchemlett.5b00120 PMC4601055

[bib20] Zhao G, Shi A, Fan Z, Du Y. Salidroside inhibits the growth of human breast cancer in vitro and in vivo. Oncol Rep 2015; 33: 2553–2560. 25814002 10.3892/or.2015.3857

[bib21] de la Cruz JF, Vergara EJ, Cho Y, Hong HO, Oyungerel B, Hwang SG. Glehnia littoralis root extract induces G0/G1 phase cell cycle arrest in the MCF-7 human breast cancer cell line. Asian Pac J Cancer Prev 2015; 16: 8113–8117. 26745047 10.7314/apjcp.2015.16.18.8113

[bib22] Kim G, An HJ, Lee MJ, Song JY, Jeong JY, Lee JH et al. Hsa-miR-1246 and hsa-miR-1290 are associated with stemness and invasiveness of non-small cell lung cancer. Lung Cancer 2016; 91: 15–22. 26711929 10.1016/j.lungcan.2015.11.013

[bib23] Taherian A, Mazoochi T. Different expression of extracellular signal-regulated kinases (ERK) 1/2 and phospho-Erk proteins in MBA-MB-231 and MCF-7 cells after chemotherapy with doxorubicin or docetaxel. Iran J Basic Med Sci 2012; 15: 669–677. 23493035 PMC3586869

[bib24] Kong Y, Chen J, Zhou Z, Xia H, Qiu MH, Chen C. Cucurbitacin E induces cell cycle G2/M phase arrest and apoptosis in triple negative breast cancer. PLoS ONE 2014; 9: e103760. 25072848 10.1371/journal.pone.0103760 PMC4114842

[bib25] Murphy KM, Ranganathan V, Farnsworth ML, Kavallaris M, Lock RB. Bcl-2 inhibits Bax translocation from cytosol to mitochondria during drug-induced apoptosis of human tumor cells. Cell Death Differ 2000; 7: 102–111. 10713725 10.1038/sj.cdd.4400597

[bib26] Levine AJ. p53, the cellular gatekeeper for growth and division. Cell 1997; 88: 323–331. 9039259 10.1016/s0092-8674(00)81871-1

[bib27] Chehab NH, Malikzay A, Stavridi ES, Halazonetis TD. Phosphorylation of Ser-20 mediates stabilization of human p53 in response to DNA damage. Proc Natl Acad Sci USA 1999; 96: 13777–13782. 10570149 10.1073/pnas.96.24.13777 PMC24141

[bib28] Oda K, Arakawa H, Tanaka T, Matsuda K, Tanikawa C, Mori T et al. p53AIP1, a potential mediator of p53-dependent apoptosis, and its regulation by Ser-46-phosphorylated p53. Cell 2000; 102: 849–862. 11030628 10.1016/s0092-8674(00)00073-8

[bib29] Rafehi H, Orlowski C, Georgiadis GT, Ververis K, El-Osta A, Karagiannis TC. Clonogenic assay: adherent cells. J Vis Exp 2011; 49: pii: 2573. 10.3791/2573 PMC3197314 21445039

[bib30] Yakisich JS, Azad N, Kaushik V, Iyer AKV. Cancer Cell Plasticity: Rapid Reversal of Chemosensitivity and Expression of Stemness Markers in Lung and Breast Cancer Tumorspheres. J Cell Physiol 2016; e-pub ahead of print 7 December 201610.1002/jcp.25725. 10.1002/jcp.25725 PMC5444977 27925198

