# Supplementary figures and images for: Antitumor effects of naturally occurring cardiac glycosides convallatoxin and peruvoside on human ER+ and triple-negative breast cancers
Source: Cell Death Discov. 2017 Feb 27;3:17009–. doi: 10.1038/cddiscovery.2017.9 (PMC5327615; doi:10.1038/cddiscovery.2017.9)

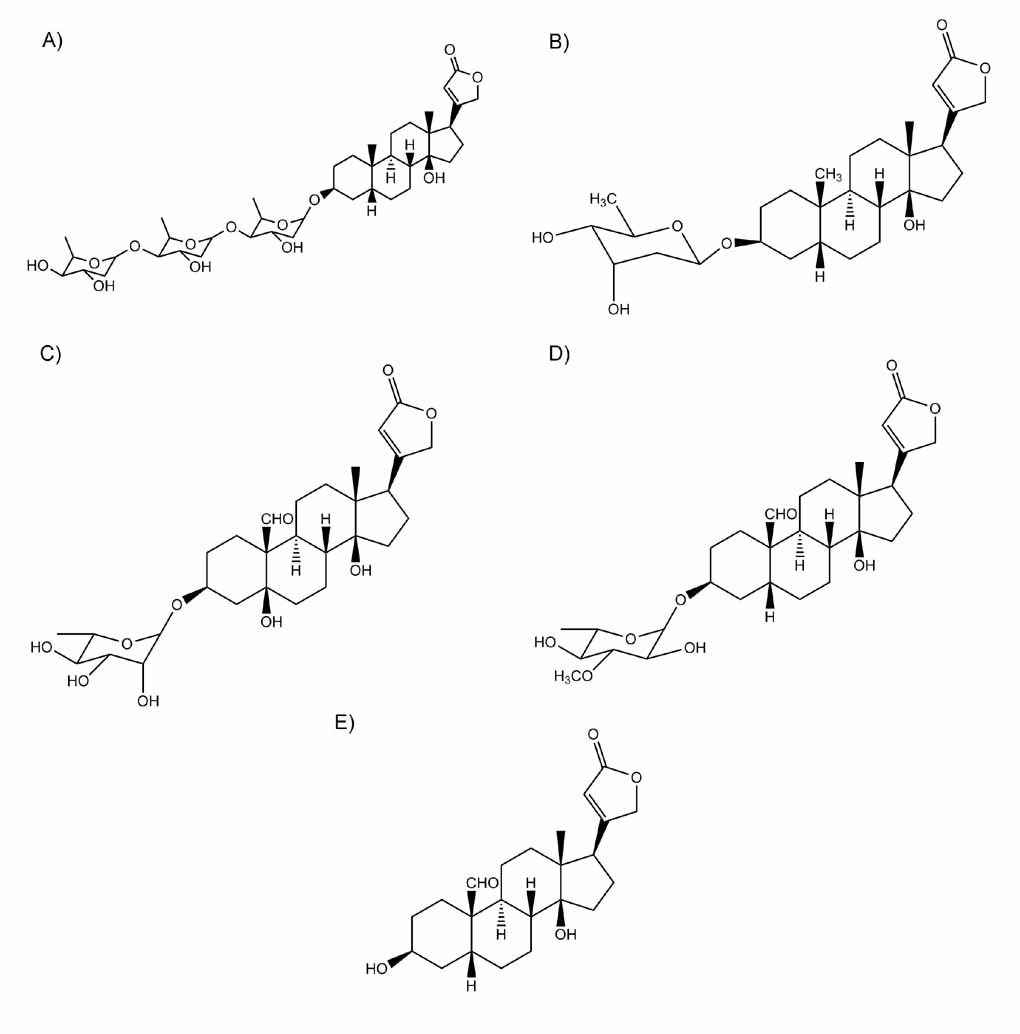

Supplement: Supplementary Figure S1 [file cddiscovery20179-s1.jpg]

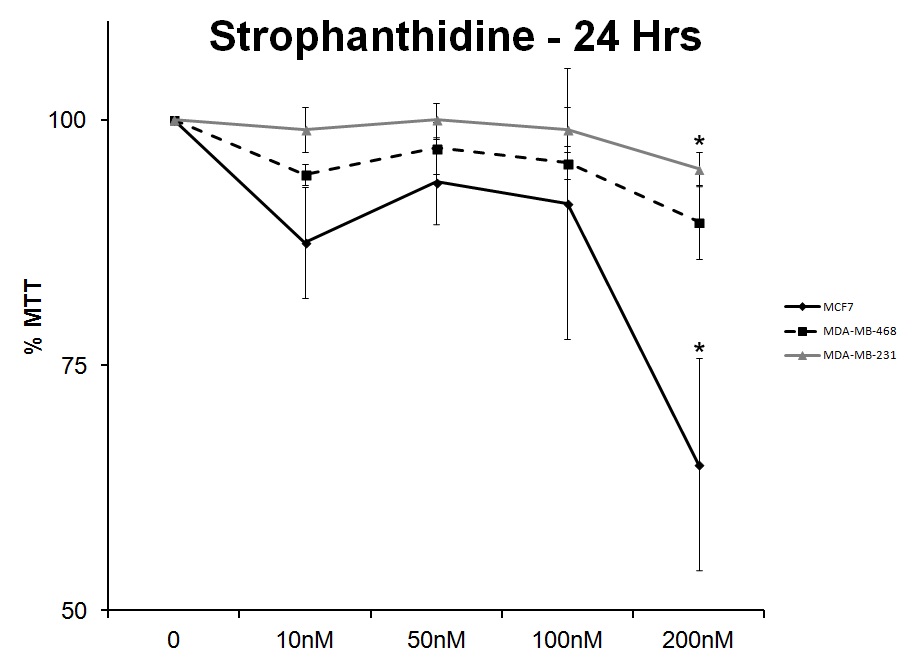

Supplement: Supplementary Figure S2 [file cddiscovery20179-s2.jpg]
